# Comparative evaluation of pain rating scales for dental pain among the Saudi population: A cross-sectional study

**DOI:** 10.1097/MD.0000000000040360

**Published:** 2024-11-08

**Authors:** Khalil Ibrahim Assiri, Shaik Mohamed Shamsudeen, Muhammed Ajmal, Abdulaziz Mustafa Asiri, Muhannad Zarbah, Saeed Abdullah Arem, Sandeepa Chalikkandy, Ali Mosfer Alqahtani

**Affiliations:** a Department of Diagnostic Science and Oral Biology, King Khalid University, Abha, Saudi Arabia; b Ministry of Defense, Armed Forces Hospital in Southern Region, Khamis Mushait, Saudi Arabia; c Ministry of Health, Saudi Arabia.

**Keywords:** dental pain, pain severity, visual analogue scale, Wong Baker facial pain perception scale

## Abstract

Pain, being a subjective phenomenon, is perceived in different manner by individuals based on various factors including age and gender. Various scales are available in literature to assess and record the pain perceived by an individual. Comparison of commonly used pain perception scales among Saudi Arabian population is scarce. A cross-sectional observational study was conducted among 180 subjects who were belonging to 2 age groups namely 7 to 16 years (group 1; n = 90) and 51 to 60 years (group II; n = 90). Subjects with dental pain of pulpal origin were included by employing consecutive sampling. The included participants were asked to record the severity of pain using visual analogue scale (VAS) and Wong Baker facial pain rating scale (WBS) in a computer-generated random order. The data obtained was subjected to statistical analysis. *t* test was used to compare the pain score recorded using VAS and WBS among males and females. The correlation between VAS and WBS was also assessed. *P* value < 0.05 was considered statistically significant. All subjects who participated responded to both the pain perception scales. No difference was found between males and females in the pain perception recorded using VAS and WBS in both the groups. A strong positive correlation was found between VAS and WBS score which was found to be statistically significant in both the groups. Both VAS and WBS are reliable tool to record pain perception in both age groups. WBS is found to be easier and more convenient one.

## 1. Introduction

The documentation of pain scores is considered to be suboptimal and forms as an important part of triage.^[1]^ Many scales does exist in documenting the intensity of pain.^[2]^ The visual analog scale (VAS) is considered to be a valid and reliable tool for measurement of all types of pain.^[3,4]^ This scale quantifies pain through 100 mm scale graduated from 0 to 100, with more scores indicating more pain.^[3,4]^ Wong Baker faces pain rating scale (WBS) is another tool which is also widely accepted due to their validity and reliability.^[5]^ WBS is a 6-point scale which consists of 6 faces ranging from a crying face to a smiling face for indicating the pain.^[5]^

For more than 2 decades, assessment of pain among pediatric population was debated in scientific literature and many studies have compared VAS and WBS.^[5,6]^ In a study conducted by Bailey et al (2007),^[1]^ it was proved that VAS had better agreement than WBS among children with abdominal pain. However, with respect to dental pain contrasting results were obtained.^[6]^ In the study conducted by Khatri et al (2012), it was proved that WBS was more sensitive than VAS.^[6]^ Many studies have been conducted to compare different pain assessment scales in dentistry^[7–10]^ however they have not compared VAS and WBS in specific.

On the other hand, pain is a perception and it can be perceived differently by people of different age groups. Hence, the age factor should be considered during assessment of the properties of the pain scales. Only limited evidences are available regarding comparison of pain scales in a wide range of age.^[10]^ Also, it is imperative to compare the 2 most commonly used scales VAS and WBS among different age groups to suggest a more reliable tool for researchers and clinicians. Thus, the aim and objectives of the present study were: (1) To compare the 2 pain scales VAS and WBS of acute dental pain among the children and elder age groups of Saudi population. (2) To determine the correlation of pain scale VAS with WBS.

## 2. Materials and methods

A cross-sectional study was conducted among patients reporting to the out-patient block of tertiary Dental Hospital in Abha, Saudi Arabia. The subjects were recruited over a period of 1 year between 2021 and 2022. The study protocol was scrutinized and approved by the Institutional Review Board bearing the reference number SRC/ETH/2018-19/13. The study participants were informed about the purpose of the study in vernacular language. Subjects who signed an informed consent form voluntarily for themselves/their child were included in the study. Verbal assent was recorded from children after obtaining parental consent.

### 2.1. Selection criteria

The study participants were recruited by following consecutive sampling technique. Subjects who reported to the outpatient department with dental ailment were screened for the eligibility criteria. Subjects with dental pain of pulpal were alone included. Subjects with physical/psychological disability, referred pain to oro-facial structure, uncooperative patients and patients with neurological dysfunction were excluded from the study.

### 2.2. Sample size estimation

Sample size was estimated using G*Power software. The input for sample size estimation included alpha error of 5%, power of 80% and beta error of 20%. By fixing the expected effect size at 25%, the sample size for the study was calculated as 40 per group. Anticipating partial/incomplete responses, the sample size for the study increased by 10% leading to a sample size of 45 per group. Ninety subjects belonging to each of the 2 age groups, namely between 7 to 16 years (group 1) and 51 to 60 years (group 2) were recruited for the study with equal representation from either gender.

### 2.3. Study protocol

The selected participants were made to sit comfortably in a dental chair. A brief case history was recorded for each participant with a focus on the chief complaint and its detailed history. For children who were unable to answer by themselves, the history was recorded from their parents. Clinical oral examination followed by intraoral periapical radiographs were made to confirm the pulpal origin of dental pain.

**Figure 1.**
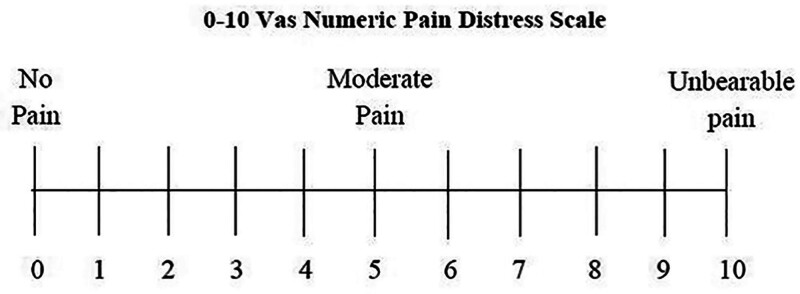
Visual analogue scale.

**Figure 2.**
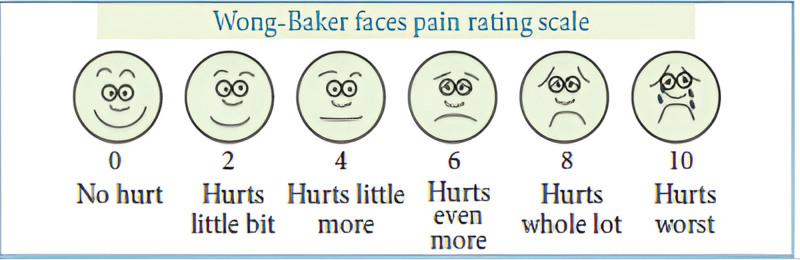
Wong Baker Faces pain rating scale.

All subjects were requested to record the severity of the perceived pain using both methods, namely visual analogue scale and Wong Baker faces rating scale in a random order which was generated by the computer to prevent order effect. The VAS is a 10 mm straight line with anchors which describe the severity of pain perception (Fig. 1). Subjects were asked to make a mark on the line that represented the present level of perceived pain intensity. WBS pain rating scale is characterized by 6 facial expressions that are descriptive of the intensity of pain in an increasing order from left to right with a numerical value of 0 to 10 indicated on the scale below it (Fig. 2). The subjects were asked to choose the face that best describes their pain perception. Each subject was explained that each face on the scale describes the level of pain ranging from none to worst.

### 2.4. Statistical analysis

All the statistical analyses were done using SPSS (Version 23, IBM, Chicago). The data obtained was checked for normality using the Shapiro–Wilk test and was found to be normally distributed. Descriptive statistics were calculated using mean, standard, percentage and frequency. Differences in VAS and WBS scores between subjects of either gender as well as age group were assessed for statistical significance using Student *t* test. The correlation in the perceived pain as recorded using either of the scales was assessed using Spearman correlation test. For all analyses, *P* value of ≤ 0.05 was considered statistically significant.

## 3. Results

A total of 660 subjects were screened for eligibility, of which 226 subjects met the inclusion criteria. Excluding the subjects who did not give consent to participate in the study, 180 subjects were included in the present study. Among the included subjects, 90 subjects belonged to the age group of 7 to 16 years with equal distribution of boys and girls while the remaining 90 subjects belonged to the age group of 51 to 60 years with equal distribution of males and females. All 180 participants belonging to either group responded to both the pain perception scales giving a response rate of 100%.

**Figure 3.**
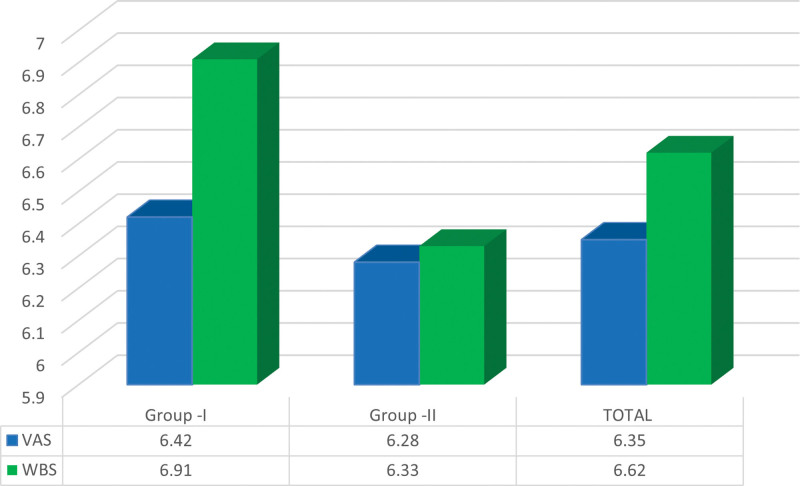
Mean VAS and WBS scores. VAS = visual analogue scale, WBS = Wong Baker pain rating Scale.

Among subjects in group 1, the mean VAS score was 6.42 ± 1.77 while, the mean WBS score was 6.91 ± 1.75. In group II, the mean VAS was 6.28 ± 2.00 while the mean WBS was 6.33 ± 2.07 (Fig. 3). In group 1, the mean VAS score of males was 6.44 ± 1.55 while that of females was 6.40 ± 1.99 while the corresponding figures for group 2 were 5.93 ± 1.92 and 6.62 ± 2.04, respectively. However, these differences were not statistically significant (Table 1).

**Table 1:** Gender wise comparison of VAS score between the groups.

Group	Males (n = 45)	Females (n = 45)	*P* value
Group-I	6.44 ± 1.55	6.40 ± 1.99	.91, NS
Group-II	5.93 ± 1.92	6.62 ± 2.04	.10, NS
Total	6.19 ± 1.75	6.51 ± 2.01	.25, NS

Student *t* test.

NS = non-significant, VAS = visual analogue scale.

**Figure 4.**
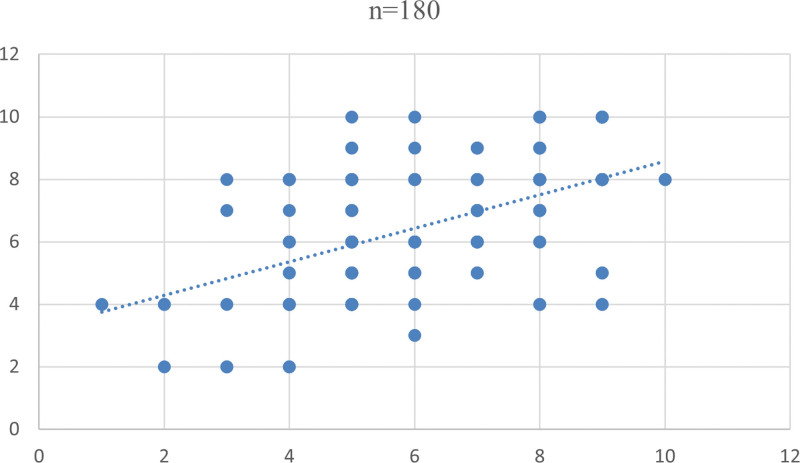
Correlation between VAS and WBS. VAS = visual analogue scale, WBS = Wong Baker pain rating Scale.

The mean WBS score of males in group 1 was 7.11 ± 1.77 while for females it was 6.71 ± 1.71. In group 2, the mean WBS score of males and females were 6.53 ±  2.29 and 6.13 ± 1.83. These differences in group 1 and group 2 were also statistically not significant (Table 2). Correlation among the pain scales were analyzed and found that there was a strong positive correlation between VAS score and WBS score which was statistically significant in both group I and II (Fig. 4) with rho values 0.69 and 0.79, respectively (Table 3).

**Table 2:** Gender wise comparison of WBS score between the groups.

Group	Males (n = 45)	Females (n = 45)	*P* value
Group-I	7.11 ± 1.77	6.71 ± 1.71	.28, NS
Group-II	6.53 ± 2.29	6.13 ± 1.83	.36, NS
Total	6.82 ± 2.06	6.42 ± 1.79	.17, NS

Student *t* test.

NS = non-significant, WBS = Wong Baker pain rating Scale.

**Table 3:** Correlation between VAS and WBS scales.

	Group-I WBS	Group-II WBS	Total WBS
Group-I VAS	*R* = 0.691 (*P* = .001*)	–	–
Group-II VAS	–	*R* = 0.794 (*P* = .001*)	–
Total VAS	–	–	*R* = 0.742 (*P* = .001*)

Spearman rank order correlation test.

VAS = visual analogue scale, WBS = Wong Baker pain rating Scale.

* *P* value < .05 statistically significant.

## 4. Discussion

Pain is considered as a fifth vital sign and has always been an important reason for patients to seek dental care.^[11,12]^ Hence, pain assessment is considered to be an integral part during diagnosis and treatment planning. However, the measurement of pain using pain assessment scales is affected by various factors such as perception of patient, age, gender and socio-economic status.^[13–15]^ The present study was conducted to assess the correlation between 2 different scales used to record pain intensity namely visual analogue scale and Wong Baker facial scale among subjects belonging to 2 different age groups.

While numerous studies have been conducted to assess the reliability of different pain rating scales in children, none have been conducted among adults especially those who are close to geriatric age group in Saudi Arabian population. The Wonk Baker faces pain rating scale is often used in Children and elders as this scale is easy to understand when compared to other scales and hence precludes the chances of overestimating the pain threshold. Similarly, the VAS is a commonly used scale to measure pain as it is objective, sensitive and reliable method to record pain perceived by an individual.

There was no difference in the pain perception between males and females using both VAS and WBS pain scale. Further, a strong correlation was found between the scores of both scales which is in concordance to the findings of among Indian population.^[6]^ This study thus provides empirical evidence for the usage of both VAS as well as WBS scales in children and adults in Saudi Arabia.

There were many other scales such as Short Version of Spielberger State-Trait Anxiety Inventory,^[16]^ McGill pain questionnaire^[17]^ and numerical rating scale^[18]^ used for pain assessment in dental clinical settings. However, to use such scales patients must be literate and need to have good comprehension on language to report their perceived pain. On the other hand, VAS and WBS are completely visual and self-descriptive, thus making it easy for patients to report their pain. The above 2 scales also provides an advantage of recording pain among illiterate and uneducated population.

Older adults due to their systemic illness and other complications perceive dental pain more differently than others.^[19]^ On the other hand, children and young adults due to their limited psychosocial maturity will be unable to make decisions on their own and this could probably affect their pain perception and reporting.^[20]^ Hence, the present study concentrated only on children and young adults of 7 to 16 years and older adults of 51 to 60 years of age to study any age-related differences in pain perception. This is in line with the findings of an Indian study conducted among 3 to 14 year old children attending the out-patient department of a dental hospital which concluded a correlation between VAS and WBS.^[6]^ Despite the correlation between the 2 scales, previous studies have shown that WBS is preferred over VAS especially among children and adolescents due to perceived difficulty in using VAS by children.^[21]^

Gender wise result showed that communication ability of males and females was similar in all age groups. Interaction between the gender and age group is not significant both for VAS and WBS. This is in consistent with the results of Ware et al.^[22]^ Our study data provides a positive correlation between the 2 pain scales. Hence the self-reporting of pain using VAS and WBS were appropriate tools for dental pain assessment in both the children as well as elders. Similar results were found in the studies conducted in the children by Garra et al^[5]^ and Briggs et al^[23]^ and found that WBS is more easier to conduct rather than VAS.

Our study results showed the positive correlation of WBS with VAS in the elder group further supports the usage of WBS in the elders for pain assessment. WBS was found most simple and preferred pain rating scale in the study conducted by Basheer.^[24]^ Kim et al also found through their study that appropriateness of faces scale for elders in the pain assessment in routine clinical practice.^[25]^

By saying this, it should also be accepted that exclusion of adults and middle aged people as one of the limitations of the study. However, considering young adults and older people as more vulnerable population, a special attention should be given during pain assessment and hence no other age groups were included in the present study.

Since, this study was carried out on out-patients visiting a dental hospital with chief complaint of pain, the chances of varied responses of pain perception has been prevented. Pain is a subjective feeling and differs based on each individual. Hence, proper measures need to be taken before comparing the scales as inconsistent responses might affect the results and hinder comparisons. Hence, rather than selecting samples randomly from general population, the study followed consecutive sampling technique where only those patients who experienced pain were recruited.

There were few short comings in the present study. First, the property of sensitivity was not taken into consideration. Sensitivity is a property of a scale to detect any small changes among population with time. Changes in pain scores post-intervention or posttreatment must be studied and compared in future to determine a more sensitive scale in clinical practice. Secondly, other factors affecting pain perception such as level of education, occupation, geographic location, and climatic conditions, etc was not considered in the study. Since, the present study primarily aimed to evaluate and compare 2 pain scales on 2 different age groups, the above factors were not considered. The last limitation might be the sampling methodology as samples were collected only from 1 site, thus affecting the representativeness and coverage of study population.

Prospective and experimental studies considering other factors such as level of education and occupation must be conducted in future. Also, it is recommended to conduct such studies in a large sample size covering a wide range of population to extrapolate the results to general population. However, based on the present study’s results, both scales are recommended to be used in clinical practice as they might be very useful in diagnosis and treatment planning.

## 5. Conclusion

From the study results, it can be concluded that both VAS and WBS are reliable tool to record pain perception in both children and older adults. Positive correlation of WBS with VAS has implications for research on pain management using the WBS as an assessment tool.

## Acknowledgments

The authors extend their appreciation to the Deanship of Scientific Research at King Khalid University for funding this work through General Research Project grant number GRP/42/45.

## Author contributions

**Conceptualization:** Khalil Ibrahim Assiri, Shaik Mohamed Shamsudeen, Muhammed Ajmal.

**Data curation:** Muhammed Ajmal, Muhannad Zarbah, Sandeepa Chalikkandy.

**Formal analysis:** Shaik Mohamed Shamsudeen, Sandeepa Chalikkandy.

**Investigation:** Abdulaziz Mustafa Asiri, Muhannad Zarbah, Saeed Abdullah Arem.

**Methodology:** Khalil Ibrahim Assiri, Abdulaziz Mustafa Asiri, Sandeepa Chalikkandy.

**Resources:** Saeed Abdullah Arem, Sandeepa Chalikkandy.

**Software:** Khalil Ibrahim Assiri, Muhammed Ajmal, Abdulaziz Mustafa Asiri.

**Supervision:** Khalil Ibrahim Assiri, Shaik Mohamed Shamsudeen, Saeed Abdullah Arem, Ali Mosfer Alqahtani.

**Validation:** Khalil Ibrahim Assiri, Muhannad Zarbah, Saeed Abdullah Arem.

**Writing – original draft:** Shaik Mohamed Shamsudeen, Muhammed Ajmal, Ali Mosfer Alqahtani.

**Writing – review & editing:** Saeed Abdullah Arem, Ali Mosfer Alqahtani.
